# The bionomics of the malaria vector *Anopheles farauti* in Northern Guadalcanal, Solomon Islands: issues for successful vector control

**DOI:** 10.1186/1475-2875-13-56

**Published:** 2014-02-15

**Authors:** Hugo Bugoro, Jeffery L Hii, Charles Butafa, Charlie Iro’ofa, Allen Apairamo, Robert D Cooper, Cheng-Chen Chen, Tanya L Russell

**Affiliations:** 1 National Vector Borne Disease Control Programme, Ministry of Health, Honiara, Solomon Islands; 2 Malaria, Other Vector-Borne and Parasitic Diseases, Regional Office for the Western Pacific, World Health Organization, San Lazaro Hospital Compound, Manila, Philippines; 3 Australian Army Malaria Institute, Gallipoli Barracks, Enoggera 4052, Australia; 4 Institute of Microbiology and Immunology, National Yang-Ming University, No 155, Sec 2, Li-Nong Street, Taipei 112, Taiwan; 5 Faculty of Medicine, Health and Molecular Sciences, Queensland Tropical Health Alliance, James Cook University, Cairns 4870, Australia

**Keywords:** Solomon Islands, Parity, Endophagy, Biting profile, *Anopheles farauti*

## Abstract

**Background:**

The north coast of Guadalcanal has some of the most intense malaria transmission in the Solomon Islands. And, there is a push for intensified vector control in Guadalcanal, to improve the livelihood of residents and to minimize the number of cases, which are regularly exported to the rest of the country. Therefore, the bionomics of the target vector, *Anopheles farauti*, was profiled in 2007–08; which was after 20 years of limited surveillance during which time treated bed nets (ITNs) were distributed in the area.

**Methods:**

In three villages on northern Guadalcanal, blood-seeking female mosquitoes were caught using hourly human landing catches by four collectors, two working indoors and two outdoors, from 18.00-06.00 for at least two nights per month from July 2007 to June 2008. The mosquitoes were counted, identified using morphological and molecular markers and dissected to determine parity.

**Results:**

Seasonality in vector densities was similar in the three villages, with a peak at the end of the drier months (October to December) and a trough at the end of the wetter months (March to May). There was some variability in endophagy (indoor biting) and nocturnal biting (activity during sleeping hours) both spatially and temporally across the longitudinal dataset. The general biting pattern was consistent throughout all sample collections, with the majority of biting occurring outdoors (64%) and outside of sleeping hours (65%). Peak biting was 19.00-20.00. The proportion parous across each village ranged between 0.54-0.58. Parity showed little seasonal trend despite fluctuations in vector densities over the year.

**Conclusion:**

The early, outdoor biting behaviour of *An. farauti* documented 20 years previously on north Guadalcanal was still exhibited. It is possible that bed net use may have maintained this biting profile though this could not be determined unequivocally. The longevity of these populations has not changed despite long-term ITN use. This early, outdoor biting behaviour led to the failure of the eradication programme and is likely responsible for the continued transmission in Guadalcanal following the introduction of ITNs. Other vector control strategies which do not rely on the vector entering houses are needed if elimination or intensified control is to be achieved.

## Background

Malaria remains a major public health issue in the Solomon Islands [1,2]. Attempts at eradication in the 1960s and 1970s greatly reduced malaria incidence but the programme was abandoned when it was realized that countrywide eradication was not obtainable after the main vector, *Anopheles farauti*, developed behavioural resistance [3,4]. With the collapse of vector control, transmission rates resurged until insecticide treated nets (ITN) were introduced in 1992–1993 [5]. This intervention measure resulted in a reduction in malaria cases from a high of 450 cases per 1000 people in 1992 to 150 cases per 1000 people in 1999 [6]. More recently, in 2008, the Solomon Islands government refocused the National Vector Borne Disease Control Programme (NVBDCP) to implement intensified countrywide control and regional elimination with financial backing from the Global Fund and AusAID. The key vector control tools are again insecticide treated nets (long-lasting insecticidal nets) and indoor residual spraying (IRS) with pyrethroids rather than DDT. This rejuvenated programme further reduced the countrywide incidence of malaria to 48 cases per 1,000 population in 2011 [1]. However, there exist large variations in malaria incidence between and within provinces [2,6,7]. One of the most malarious areas in the country is Guadalcanal Province [1] which has historically been a problem area [8] and where in 2011 there were 87 cases per 1000 people (Ministry of Health, unpublished data).

Understanding the behaviour of the local vectors is essential for planning vector control activities. The primary vector control tools, ITN and IRS, depend on mosquitoes biting indoors, late in the night and resting indoors after feeding. There are three species of malaria vectors in the Solomon Islands: *Anopheles punctulatus*, *Anopheles koliensis* and *Anopheles farauti*[9]. The malaria eradication programme of the 1960-70s had reduced the distribution and abundance of *An. koliensis* and *An. punctulatus*, both of which were late night and highly endophagic biters [4], leaving *An. farauti* as the primary vector of malaria. The bionomics of *An. farauti* in Guadalcanal Province was profiled in the early 1990s, prior to the introduction of ITNs. At this time, *An. farauti* occurred in large numbers, with peak biting outdoors and early in the night (21.00) and the entomological inoculation rates (EIR) was up to 1,022 infective bites/person/year [10-12]. Recent work in the elimination provinces of the Solomon Islands indicates that this early night, outdoor feeding pattern is still maintained [13,14]. Such early night outdoor feeding behaviour of *An. farauti* could potentially compromise the efficacy of the vector control programme.

The NVBDCP is driven to reduce malaria transmission in Guadalcanal to improve the livelihood of the residents, but also because large numbers of cases are continually exported to the elimination provinces. The area around Red Beach and Koli Point, about 20 km east of Honiara, was used extensively in the late 1980s to early 1990s to study the bionomics of *An. farauti* and to trial the comparative effectiveness of DDT-IRS and pyrethroid ITN [5,10-12,15]. More recent faunal surveys have verified that *An. farauti* remains very common along the north coast of Guadalcanal [16,17]; however for 20 years no studies profiled the bionomics of the vector. During this time frame ITNs were introduced into the area (in 1992–1993) and distribution and re-treatment activities were completed annually by the NVBDCP (Ministry of Health, unpublished data). The annual coverage rates varied depending on the availability of funds and political stability; nonetheless there was a continual presence of ITNs in the area. The hypothesis for this study questioned if the modified feeding behaviour of *An. farauti* observed after the use of DDT-IRS had been maintained over time. Such information is fundamental to conducting successful elimination or intensified control programmes.

## Methods

### Study site

**Figure 1:**
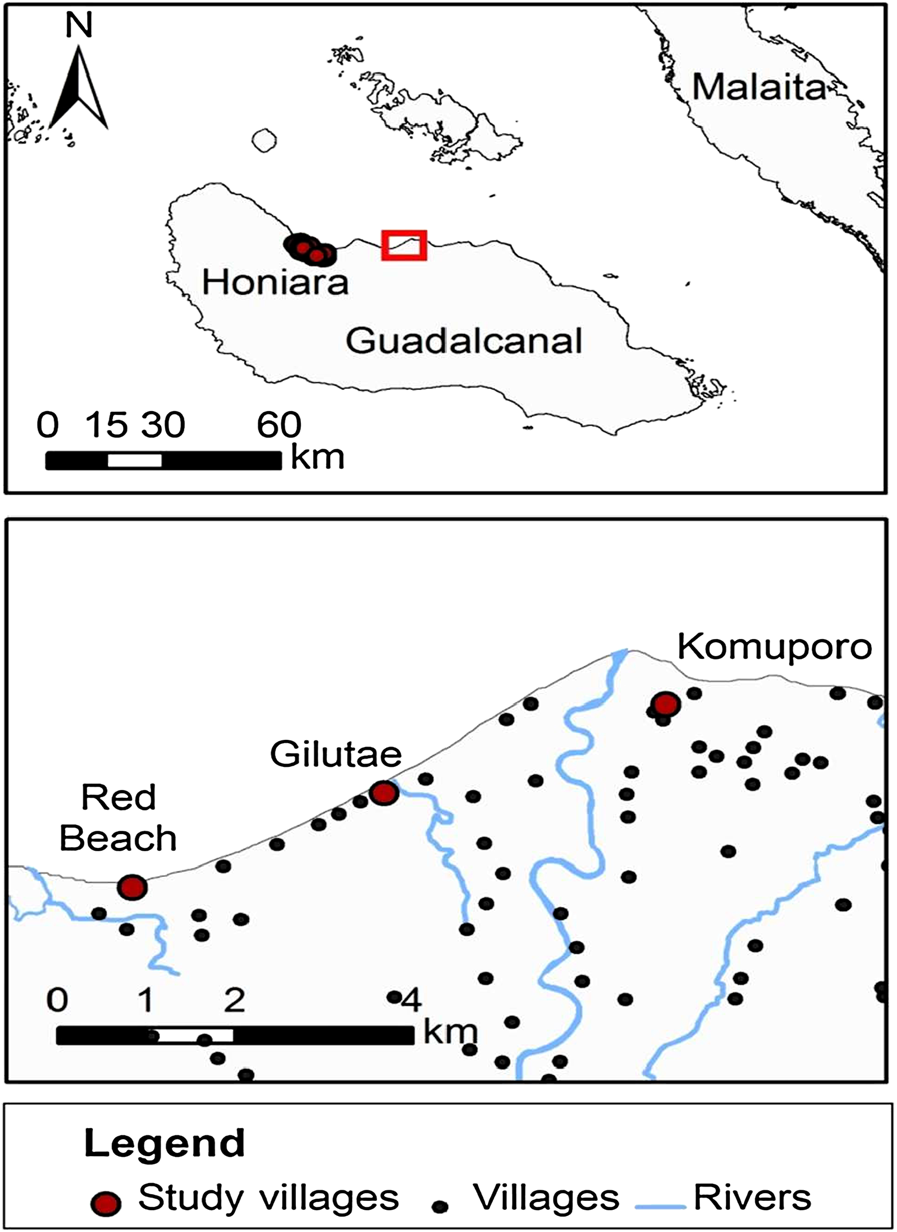
**Map showing the location of the study villages.** Top: Guadalcanal Island. Bottom: North Guadalcanal showing the three study villages and their proximity to other villages.

The study was conducted in northern Guadalcanal in three coastal villages: Red Beach (E, 160°06.872′; S, 09°25.791′), Gilutae (E, 160°07.957′; S, 09° 25.206′) and Komuporo (E, 160°09.771′; S, 09°24.755′) (Figure 1). The study area is 20 km east of the capital Honiara and encompasses numerous rural villages scattered throughout bushland on a low lying coastal plain that is cut by numerous creeks and rivers, which end on the coast in brackish swamps and lagoons. The climate of the region is continuous hot wet with an annual rainfall of 2,500 mm (median of 20 years) [18]. Rain falls throughout the year, however there is some seasonality with the months December to May receiving higher rainfall than June to November. The mean annual temperature on the coast is 26°C and is constant throughout the year with daily fluctuations greater than any annual fluctuations.

### Human landing catches (HLC)

Seasonality, biting densities and biting behaviour were ascertained by human landing catches (HLC) conducted from 18.00-06.00 at least twice monthly from July 2007 to June 2008 in each village. Sampling was not conducted in February 2008 due to flooding. Catches were made by four collectors, two working indoors and two working outdoors, for 40 min each hour; all mosquitoes coming to bite the collectors’ exposed feet and legs were caught using a torch and aspirator. The first team of four collectors worked from 18.00 to 00.00 and the second team of four collectors worked from 00.00 to 06.00. Mosquitoes were held in individual waxed paper cups for each hour and location. The collectors were rotated through the collection sites to compensate for differences in individual odours and collecting abilities. The following morning, mosquitoes were killed, counted and dissected to determine parity [19]. All *Anopheles* were identified by morphology [20]. Specimens were desiccated and preserved on silica gel. A subset of the specimens collected were identified by molecular analysis of the Internal Transcribed Spacer Region II of the ribosomal DNA [21] on a subset of the entire collection. In Guadalcanal, *An. farauti* has been shown to be the only member of the *An. farauti* complex which feeds on humans [9], hence the subset was analysed to confirm this.

### Biting behaviour

The biting behaviour of *An. farauti* was compared using: 1) propensity to bite indoors (endophagy), and 2) propensity to bite during sleeping hours (nocturnal biting). The degree of endophagy was calculated as the proportion of mosquitoes biting indoors as follows:

$$I_{18.00\to 05.00}/\left(I_{18.00\to 05.00}+O_{18.00\to 05.00}\right)$$

where *I* = the total number of mosquitoes caught indoors, *O* = the total number of mosquitoes caught outdoors and the subscripts represent the start time for each hour [22]. Nocturnal biting was calculated as the proportion of mosquitoes biting either indoors or outdoors during peak sleeping hours (hours starting 9 pm to 4 am) as follows [22]:

$$\left(I_{21.00\to 04.00}+O_{21.00\to 04.00}\right)/\left(I_{18.00\to 05.00}+O_{18.00\to 05.00}\right).$$

### Survival

The ovaries of mosquitoes caught in the night landing catches were dissected in physiological saline, allowed to dry and examined under 100-200X for the presence or absence of skeins at the end of the trachea [19]. From this the proportion parous (P) was used to determined the survival through one day (*p*) as ^*x*^√P; where *x* is the length of the gonotrophic cycle [23].

### Statistics

The dataset was constructed with two tables: 1) field collections, and 2) parity dissections [24]. Statistical differences in mosquito biting rates between the study villages were compared using a generalized linear mixed model (GLMM) with a negative binomial distribution and a random factor to account for sample period. The differences in endophagy, nocturnal biting and parity were determined using a binomial generalized linear model (GLM) with an explanatory variable for study period, village or collection time. All analyses were conducted using the *R* package V2.14.2.

### Ethics

For this research, ethical approval was granted from the Solomon Islands Ministry of Health for conducting human landing catch as a routine programmatic activity.

## Results

### Species identification

All mosquitoes collected by HLC (*n* = 3,405) were determined to be *An. farauti* s.l. by morphology. A subset of them were confirmed to be *An. farauti* s.s. by molecular analysis (*n* = 543 PCR amplifications).

### Seasonality and vector densities

**Figure 2:**
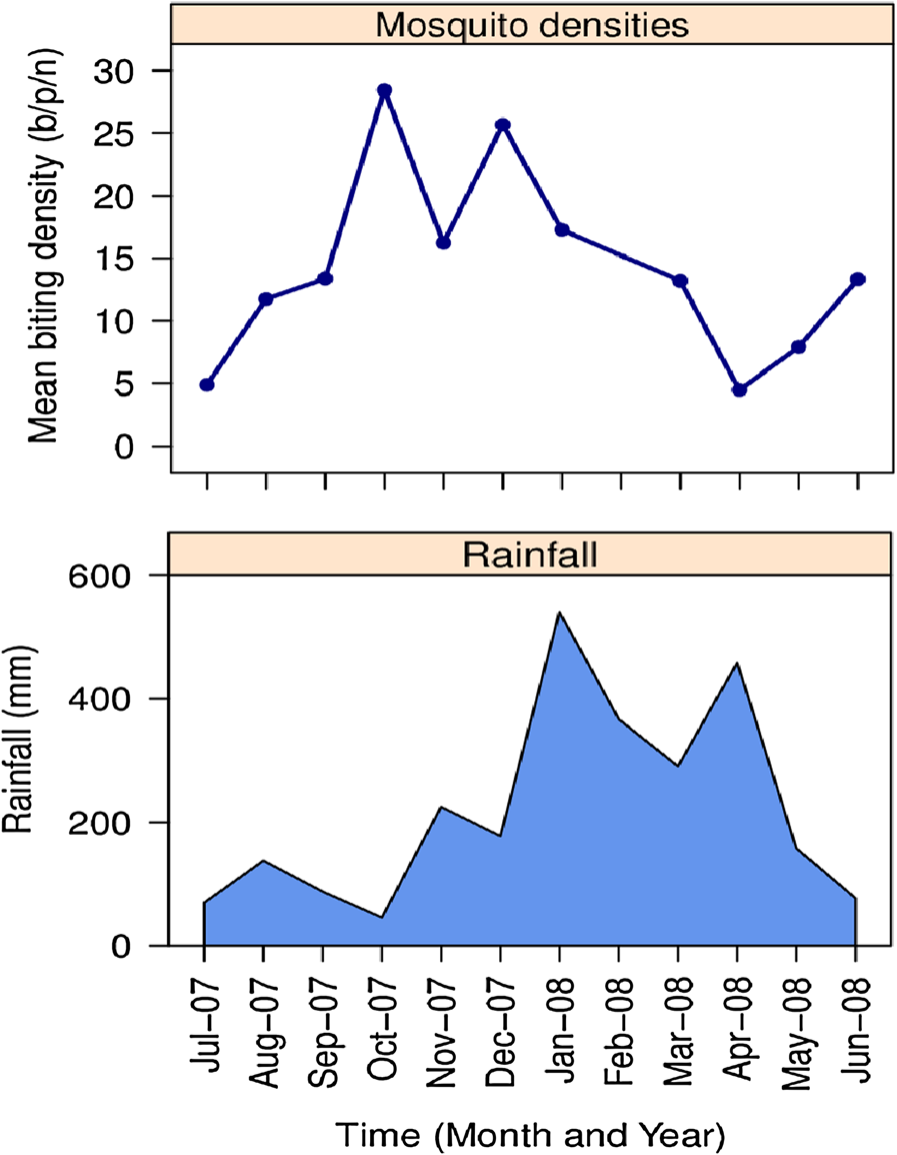
**Monthly biting rates of *Anopheles farauti. ***(Top) and rainfall (Bottom) for north Guadalcanal.

The average biting densities of *An. farauti* over the year differed between the three villages (*β* = 0.165, se = 0.077, *p* = 0.036). The biting rate at Red Beach (17.07 bites/person/night (b/p/n)) was higher than at Komporo (10.80 b/p/n) and Gilutae (12.09 b/p/n) (Table 1). These differences in biting rates reflect the variation in productivity larval sites that were available around each village [17]. The seasonal trend was the same for each village with vector densities peaking at the end of the drier months of October (56.9 b/p/n), November (32.5 b/p/n) and December (51.3 b/p/n) with the mean biting density over this three month period being 46.9 b/p/n. Vector densities fell in February to their lowest at the end of the wettest months: March (26.4 b/p/n), April (9.6 b/p/n) and May (15.8 b/p/n), with a mean biting density for this three month period of 17.3 b/p/n (Figure 2).

**Table 1:** **The entomological estimation of the feeding behaviour and survival rates of *Anopheles farauti *** **from three villages on Northern Guadalcanal, Solomon Islands during July 2007 to June 2008**

**Entomological parameters**	**Gilutae**	**Komporo**	**Red Beach**	**Overall**
Biting rate (B: b/p/n)				
Indoor	7.00	6.62	15.8	9.66
Outdoor	17.18	14.98	19.09	17.08
Overall	12.09	10.80	17.07	13.40
Endophagy^1^ (Proportion indoors ± se)	0.29 ± 0.014 (*n* = 1,064)	0.31 ± 0.015 (*n* = 907)	0.44 ± 0.013 (*n* = 1,434)	0.36 ± 0.008 (*n* = 3,405)
Nocturnal biting^2^ (Proportion 21.00-05.00 ± se)	0.31 ± 0.014 (*n* = 1,064)	0.35 ± 0.016 (*n* = 907)	0.39 ± 0.013 (*n* = 1,434)	0.35 ± 0.008 (*n* = 3,405)
Parity (Proportion parous) (n/total)	0.542 (552/1,017)	0.577 (523/906)	0.541 (764/1,412)	0.551 (1,839/3,335)

B = no. of mosquitoes collected/no. of nights/no. of collectors.

^1^Proportion of mosquitoes caught indoors calculated as: *I*_18.00 → 06.00_/(*I*_18.00 → 06.00_ + *O*_18.00 → 06.00_); where *I* = the total number of mosquitoes caught indoors, *O* = the total number of mosquitoes caught outdoors and the subscripts represent the time for each hour.

^2^Proportion of mosquitoes caught during hours when most people are asleep calculated as: (*I*_22.00 → 05.00_ + *O*_22.00 → 05.00_)/(*I*_18.00 → 06.00_ + *O*_18.00 → 06.00_).

### Biting behaviour

**Figure 3:**
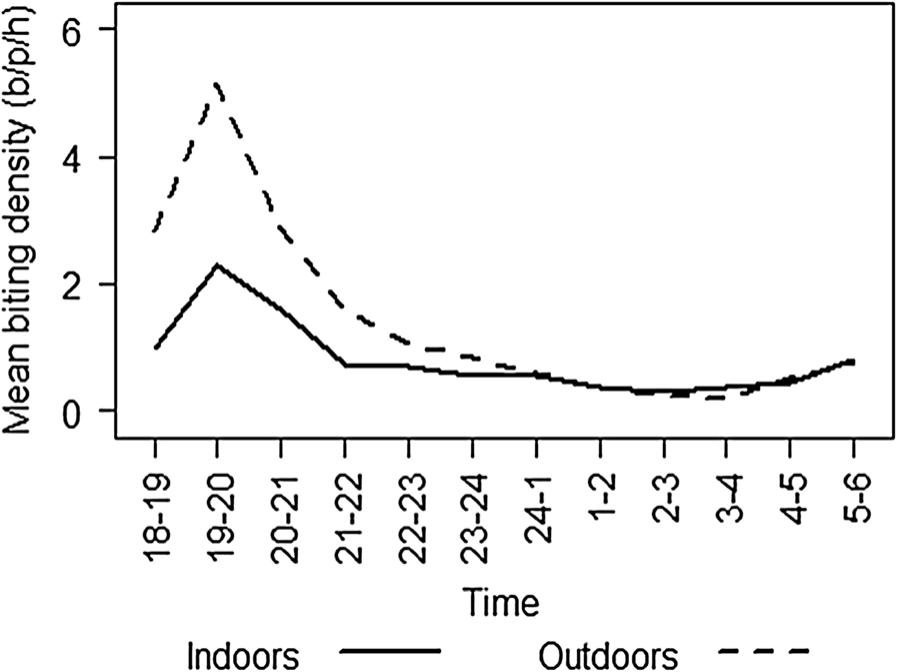
**The hourly indoor and outdoor biting profile of *Anopheles farauti *** **from 18.00 to 06.00 in north Guadalcanal.**

The degree of endophagy (indoor biting) varied over the sample periods (*β* = −0.057, se = 0.011, *p* <0.001) and also between villages (*β* = 0.372, se = 0.043, *p* < 0.001). Endophagy was 0.29 ± 0.014 for Gilutae, 0.31 ± 0.015 for Komporo and 0.44 ± 0.013 for Red Beach (Table 1). Similarly the degree of nocturnal biting (activity during sleeping hours) varied over the sample periods (*β* = 0.022, se = 0.011, *p* = 0.048) and also between villages (*β* = 0.187, se = 0.042, *p* <0.001). Nocturnal biting was 0.31 ± 0.014 for Gilutae, 0.35 ± 0.016 for Komporo and 0.39 ± 0.013 for Red Beach (Table 1). Such levels of variation in the biting profiles are reasonable in field populations, and the general biting pattern was consistent throughout all sample collections. Overall, the majority of biting occurred outside of houses (64%) and outside of sleeping hours (65%) when people are unprotected by LLINs and/or IRS. Biting commenced early in the evening (at dusk 18.30) and rose rapidly to a peak in the second hour of the night (19.00-20.00) it then declined throughout the rest of the night to a low at 02.00-03.00 (Table 2, Figure 3). A minor increase in biting activity occurred during the two hours before dawn (04.00-06.00) (Figure 3). More than half (59%) of all host seeking occurred during the first three hours of the night (18.00-21.00).

**Table 2:** **The parity and biting profile of *Anopheles farauti *** **compared for each hour of the night**

			**Mean biting rate (b/p/h)**		**Mean parous biting rate (b/p/h)**	
**Time**	**Parity**	**n/total**	**Indoor**	**Outdoor**	**Indoor**	**Outdoor**
18.00-19.00	0.433	213/492	0.984	2.823	0.426	1.222
19.00-20.00	0.517	478/925	2.302	5.125	1.189	2.648
20.00-21.00	0.574	332/561	1.587	2.875	0.911	1.650
21.00-22.00	0.567	166/293	0.746	1.586	0.423	0.899
22.00-23.00	0.581	129/222	0.683	1.078	0.397	0.626
23.00-00.00	0.663	114/172	0.548	0.828	0.363	0.549
00.00-01.00	0.678	99/146	0.548	0.594	0.371	0.403
01.00-02.00	0.670	61/91	0.357	0.375	0.239	0.251
02.00-03.00	0.623	43/69	0.310	0.242	0.193	0.151
03.00-04.00	0.767	56/72	0.381	0.211	0.292	0.162
04.00-05.00	0.550	60/109	0.444	0.523	0.245	0.288
05.00-06.00	0.538	98/182	0.794	0.781	0.427	0.421
Total	0.551	1,839/3,335	0.807	1.422	0.412	0.727

### Survival

Dissections to measure parity were made on over 100 mosquitoes each month except for July 2007 where only 39 were dissected; in all 3,335 *An. farauti* were dissected over the year. The proportion parous did not vary between villages (*β* = 0.019, se = 0.041, *p* = 0.632) and showed no seasonality over the year (*β* = −0.001, se = 0.011, *p* = 0.903). Overall the mean parity rate was 55.1% (1,839/3,335). With a gonotrophic cycle of 2.3 days [10], the probability of survival through one day was 79%.

The only heterogeneity noted was a variation in parity throughout the night (*β* = −0.032, se = 0.015, *p* = 0.032), with the first hour of the night having a lower proportion parous (43.3%) than the rest of the night. However, it is unlikely that this phenomenon would have any epidemiological relevance considering the early-biting cycle of *An. farauti*. The mean biting rate of parous mosquitoes throughout the night was calculated by adjusting the total biting rate by the proportion parous. It is evident that the majority of human exposure to parous mosquitoes occurs before 21.00.

## Discussion

This study observed a distinct seasonality in adult *An. farauti* densities. It is important that this seasonality is considered when planning the timing of vector control activities by the NVBDCP; in particular it would be advantageous if annual activities were completed before the peak in biting occurs towards the end of the year. This seasonality reflects the larval ecology of this species and its ability to utilize brackish water lagoons for oviposition [25,26]. A study of the larval ecology in the study villages was simultaneously conducted [17], which demonstrated that larval presence and density also varied seasonally and was primarily driven by rainfall. Larval abundance was highest in the drier months when brackish lagoons formed at the mouth of the streams behind sandbars. In this supporting study [17], the peak in larval abundance occurred from September to December and would have supported the higher adult densities observed at this time. When rainfall was high (January to April), the sandbars at the mouth of the streams were washed away and in the following month the density of both larvae and adults was lower. The negative association of severe rainfall with reduced larval and adult densities of *An. farauti* is supported by previous studies from both Vanuatu [27] and Papua New Guinea [28].

In the current study, the populations of *An. farauti* in Guadalcanal were observed to feed primarily outdoors and early in the evening. During the original eradication programmes of the late 1960s and early 1970s, it was observed that *An. farauti* avoided exposure to DDT-IRS by changing their feeding behaviour [29]. In Makira-Ulawa Province prior to DDT-IRS use, the percentage of *An. farauti* biting before 21.00 was around 40% and there was equal feeding indoors and outdoors [29]. After DDT-IRS was implemented, the percentage of biting before 21.00 rapidly increased to more than 70% and the majority (66%) of biting shifted to outdoors [29]. After DDT pressure was eventually removed from the mosquito populations, the modified behaviour of *An. farauti* persisted. More recently, over the past five years, this early, outdoor biting has been observed to be sustained across the country in both Temotu [13] and Isabel Provinces [14].

Previously on northern Guadalcanal, the biting behaviour of *An. farauti* was profiled in 1988 [10]. The previous study was conducted after the DDT-IRS of the original eradication programme had ceased, and also before ITNs were introduced in 1993. In 1988, the peak biting time for *An. farauti* was 21.00-22.00 and endophagy was 30% (range 16 to 32%) [10]. In the current study, conducted 20 years later, the peak biting time was earlier at 19.00-20.00 and endophagy was similar with 36% feeding indoors. As this change to early outdoor biting was already in place prior to the introduction of ITN it is not possible to state unequivocal that this behaviour has been maintained in *An. farauti* populations by the introduction of ITNs. However there is evidence that early night feeding was increased in *An. farauti* after only 3 weeks of ITN use in an area which had previously had none or very little DDT-IRS [30]. Also following the implementation of elimination efforts using ITNs and pyrethroids- IRS in Temotu Province, a lower portion of mosquitoes sought meals after 21.00 [13]. This persistence in early, outdoor biting will allow malaria to be maintained as is evident in the cases of malaria reported over the last 20 years [1]. As such there is a need to develop an integrated vector control programme which utilizes complementary strategies that consider the subtleties of mosquito ecology to further reduce the density of the local vector populations and associated malaria transmission. The most promising complementary tool which is currently available is larval control; other complementary tools generally remain at the proof of principle stage and there is a need to prioritize research funding to facilitate investigations of potential efficacy before they can be adapted into programmatic use.

Interestingly, there are indications that the behaviour of *An. farauti* inside of sprayed houses has also changed [5]. In the same area of northern Guadalcanal, the blood-feeding success and survival of *An. farauti* was assessed in 1989–1991. Of those females which entered houses, collections from exit window traps showed that about half were able to successfully obtain a blood meal in either houses sprayed with DDT (52% fed) or houses with an ITN (43% fed) [5]. However, the 24-hour mortality rates in these mosquitoes differed significantly, with 98.2% (n = 219) mortality of females collected from houses with ITN, but only 10.1% (n = 24) mortality of females collected from DDT-sprayed houses. This indicated that *An. farauti* was able to avoid the insecticides on the walls and leave immediately after obtaining the blood meal. It should be noted that as this time, *An. farauti* was susceptible to DDT and mortality when exposed in WHO susceptibility tests was 77% [5]. Additionally, the susceptibility of *An. farauti* to both DDT and pyrethroids was also assessed in 2006 and 100% mortality of wild-caught adults was recorded (Ministry of Health, unpublished data). Whether *An. farauti* will show the same behavioural response to IRS with pyrethroids is unknown.

The annual mean parous rate recorded in this study (54.8%) is similar to that recorded 20 years earlier in the area both in unsprayed (55.5%) and DDT-sprayed houses (53.6%) [5,10]. However, in 1989–1991 the parous rates in houses provided with ITN were lower (49.9%) than the sprayed and unsprayed houses [5], indicating that ITNs had an initial impact on the longevity of the *An. farauti* populations; but this was not sustained, possibly due to deterioration in net quality and insecticidal efficacy. Interestingly, this survival rate from 2007–08, recorded just prior to the introduction of LLINs, appears to be higher than that recorded in other populations of *An. farauti* in the Solomon Islands from 2008 onwards (42% in Temotu [13] and 41% in Isabel [14]). In the current study, the parous rate was relatively stable throughout the year and did not fluctuate with the changes in *An. farauti* densities. However, parity rate for the first hour of biting (18.00-19.00) was dominated by more nulliparous mosquitoes than the remainder of the night. This has similarly been seen in populations of *An. farauti* in Temotu Province [13] and Central Province (Russell *et al.* unpublished data) and *An. punctulatus* in Papua New Guinea [31]. However, for *An. farauti* it is unlikely that this phenomenon would have any epidemiological relevance. Considering the early-biting cycle, the majority of human exposure to parous mosquitoes – which are older and have taken multiple blood meals – still occurs before 21.00.

## Conclusion

The current study describes the bionomics of the primary malaria vector in the Solomon Islands, *An. farauti*. Recently, the NVBDCP of the Solomon Islands refocused to implement intensified countrywide control and regional elimination. The key to a successful programme will be understanding, and responding to, the behaviours of the target vector. It was observed that *An. farauti* has a distinct seasonal profile, with peak activity from October to December, indicating that annual vector control activities should be completed before this period. Importantly, it was observed that the early outdoor biting habit of *An. farauti*, first observed in the study area in 1988 still persists 20 years later. With this feeding behaviour, the target mosquitoes are able to minimize exposure to ITNs and IRS. Therefore, there is a need to implement complementary tools that provide personal protection or target other bionomics’ vulnerabilities that may exist outside of houses, such as in the larval stages, during mating, sugar feeding or any other aspect of the life cycle. This will not only improve the success of vector control in Guadalcanal, but will reduce the number of cases that are exported to the control provinces.

## Availability of supporting data

The datasets supporting the results of this article are available in the VecNet repository: https://dl.vecnet.org/files/db78tc04f; doi:10.7274/R0J1012M.

## Abbreviations

GLM: Generalized linear model; GLMM: Generalized linear mixed model; HLC: Human landing catch; IRS: Indoor residual spray; ITN: Insecticide-treated net; LLIN: Long-lasting insecticidal net; NVBDCP: National vector Borne Disease Control Programme.

## Competing interests

The authors declare that they have no competing interests.

## Authors’ contributions

HB, JLH, and CCC designed the study; HB, CB, CI, AA, and CCC performed the fieldwork; HB, RDC and TLR analysed the data and wrote the manuscript. All authors read and approved the final manuscript.
